# Effects of *Cyclocarya paliurus* Aqueous and Ethanol Extracts on Glucolipid Metabolism and the Underlying Mechanisms: A Meta-Analysis and Systematic Review

**DOI:** 10.3389/fnut.2020.605605

**Published:** 2020-12-01

**Authors:** Wei Liu, You Wu, Yuli Hu, Shuai Qin, Xiaoyuan Guo, Minghui Wang, Lili Wu, Tonghua Liu

**Affiliations:** ^1^Key Laboratory of Health Cultivation of the Ministry of Education, Beijing University of Chinese Medicine, Beijing, China; ^2^Dongfang Hospital, Beijing University of Chinese Medicine, Beijing, China; ^3^First School of Clinical Medicine, Guizhou University of Traditional Chinese Medicine, Guiyang, China; ^4^Chengdu Integrated TCM and Western Medicine Hospital, Chengdu, China

**Keywords:** *Cyclocarya paliurus*, metabolic syndrome, insulin resistance, dyslipidemia, hyperglycemia, glucolipid metabolism

## Abstract

**Background and Aims:** *Cyclocarya paliurus* (CP) has been used as an herbal tea to treat diabetes mellitus and obesity for hundreds of years. Previous research suggests that CP specifically restores glucolipid metabolic homeostasis, and the two most studied preparations are aqueous and ethanol extracts. In order to verify the effect of CP on glucolipid metabolism in animal models with metabolic syndrome, a meta-analysis was performed, and the active components and underlying mechanisms were systematically reviewed.

**Methods:** Four databases: PubMed, Web of Science, Embase, and Cochrane Library were searched to identify potential literature. Data of blood glucose (BG) level, area under curve (AUC) of oral glucose tolerance test (OGTT), total cholesterol (TC), triglyceride (TG), high-density lipoprotein (HDL), and low-density lipoprotein (LDL) levels were extracted as indicators of the assessment of CP's effects. Follow-up analyses including subgroup analysis, meta-regressions, and publication bias were also conducted.

**Results:** A total of 96 papers were identified from the databases and 11 papers including 31 data reports were involved in the meta-analysis. CP had a positive effect in down-regulating BG, AUC of OGTT, TC, TG, and LDL, and up-regulating HDL (*P* < 0.001, 95% confidence interval of standard mean difference did not incorporate the null value 0).

**Conclusion:** CP showed definite activity of regulating glucolipid metabolism in animal models, and it exerted its function through multiple mechanisms including but not limited to: (1) improving insulin resistance; (2) protecting pancreatic β cells; (3) decreasing inflammatory infiltration; and (4) anti-oxidative stress.

## Introduction

Metabolic syndrome, also known as syndrome X, is a complex syndrome characterized by glucose intolerance, insulin resistance, visceral obesity, dyslipidemia, and hypertension (1). It is associated with the global prevalence of diabetes mellitus and contributes to the increasing risk of cardiovascular diseases (2). The incidence of metabolic syndrome runs parallel with, but is three times more prevalent than that of type 2 diabetes, so it is estimated to influence around 25% of the global population (3).

The definition of metabolic syndrome has varied since the concept was established (4). However, in 2009, a harmonized standard was formed by five organizations (5), and this united criteria included the followed parameters: waist circumference, fasting plasma glucose level, high-density lipoprotein cholesterol (HDL-C) level, triglyceride (TG) level, and blood pressure level. Over-consumption of energy and lack of exercise are believed to be the predominant causes of metabolic syndrome. The accumulation of visceral fat is related to insulin resistance, glucose intolerance, lower HDL-C level, and higher low-density lipoprotein cholesterol (LDL-C) level and TG levels (6). So, there is no doubt that the regulation of glucolipid metabolism plays an important role in controlling metabolic syndrome.

Management strategies for metabolic syndrome are yet to be developed, and lifestyle modification and drug therapy are currently considered to be the two main approaches for its control (1). *Cyclocarya paliurus* (CP) (Batal.) Iljinsk (family Cyclocaryaceae) is an indigenous plant distributed throughout Southern China, the leaves of which have been used as an herbal tea to treat hyperglycemia and obesity in the local population (7). Recent scientific research shows that CP acts to reduce blood sugar levels and blood lipid levels, lowers body weight, and lowers blood pressure (8). Furthermore, a health tea made from CP leaves was approved by the United States Food and Drug Administration (FDA) in 1999, and was the first Chinese herbal tea approved by the institution (9, 10). In short, as a dietary supplement, CP seems to be a potential candidate for assisting in the prevention and treatment of metabolic syndrome.

To date, studies on CP have mostly involved preclinical research based on rodents. Among these studies, aqueous extract and ethanol extract have been the two most commonly studied CP preparations. Because of discrepancies in extraction processes, the components of the two extracts are different: polysaccharides are abundant in the aqueous extract (10), while the ethanol extract is enriched with flavonoids and triterpenoids (11). Polysaccharides (12), triterpenic acids (13, 14), and flavonoids (15) were initially believed to be the active constituents of CP, however, there is also a study suggesting that polysaccharides may not be the anti-diabetic component of CP (16). Moreover, some studies also suggest that *Cyclocarya paliurus* aqueous extract (CPAE) shows no significant impact on lipid metabolic profile (15, 17). This controversy could be answered by a meta-analysis that would combine current evidence and reach a more robust conclusion. Moreover, previous studies have found that CP exerts a protective effect over pancreatic β cells (8), has an anti-inflammatory effect (7, 18), and an anti-oxidant effect (16, 19) in animal models; however, the detailed mechanisms of CP's therapeutic activities still need to be unveiled.

In the current study, we performed a meta-analysis regarding the effect of CPAE and *Cyclocarya paliurus* ethanol extract (CPEE) on glucolipid metabolism with data obtained from preclinical research. Blood glucose (BG) level and the area under curve (AUC) of the oral glucose tolerance test (OGTT) are viewed as indicators of a hypoglycemic effect, while total cholesterol (TC), triglyceride (TG), high-density lipoprotein (HDL), and low-density lipoprotein (LDL) are used as indicators of a hypolipidemic effect. A systematic review was performed on the mechanisms studied by previous researchers, and we also summarized the current extraction methods for CP, and the major components of CPAE and CPEE, so as to form a comprehensive understanding of presently available articles and provide an inclusive reference for further studies.

## Methods

This meta-analysis and systematic review were conducted according to Preferred Reporting Items for Systematic Reviews and Meta-Analyses (The PRISMA Statement) (20).

### Search Strategy

Four academic databases: PubMed, Web of Science, Embase, and Cochrane Library, were searched from inception to May 2020. The PICO (patients, interventions, comparisons, outcomes) adopted in this analysis were as follows: (1) Animal models of T2DM/obesity/hyperlipidemia/metabolic syndrome. (2) CP extracts were orally administered to the animals and interventions were not combined with other chemicals or treatments. (3) The comparison group consisted of a same model that was treated with a placebo. (4) BG, OGTT, TC, TG, HDL, or LDL for the treatment and comparison groups at the end of intervention. In order to find the required articles, searches were conducted as follows: (1) search “*Cyclocarya*” or “sweet tea tree” (title/abstract/keywords/MeSH); (2) search diseases with the following terms: “diabetes,” “diabetic,” “insulin resistance,” “obesity,” “hyperlipidemia,” and “glucose intolerance” (title/abstract/keywords/MeSH); (3) terms, such as “animal,” “rat,” “mouse,” and “rodent” (title/abstract/keywords/MeSH) were used to discover animal experiments; (4) the search queries above were combined together using “AND” (Supplementary Table 1). There was no limit on language, study design, and type of publication adopted for the research results.

### Eligibility Criteria for Studies

#### Inclusion Criteria

The inclusion criteria for the title and abstract were as follows: (1) not a review/protocol/letter/meta-analysis/proceeding; (2) oral administration of CP extract or component (no combination with other chemicals or treatments); (3) animal experiments (neither clinical trials nor *in vitro* research); (4) animal models of T2DM/obesity/hyperlipidemia/metabolic syndrome.

#### Exclusion Criteria for Systematic Review

The exclusion criteria for meta-analysis were as follows: (1) lack of full-text; (2) unneeded disease model; (3) not aqueous or ethanol extract of CP.

#### Exclusion Criteria for Meta-Analysis

The exclusion criteria for meta-analysis were as follows: (1) lack of full-text; (2) unneeded disease model; (3) not aqueous or ethanol extract of CP; (4) lack of data required; (5) treatment that lasted for <2 weeks; (6) repeated data in more than one article; (7) inconsistent depiction in article.

The titles and abstracts of articles from search results were examined by two independent researchers, duplicates were eliminated, and studies that met the inclusion criteria were reserved. Then researchers screened the full-text of articles according to the exclusion criteria, and the remaining articles were considered eligible. Whenever there were disagreements, a third author was consulted.

### Data Extraction

The following data was extracted by two independent researchers: (1) publication information: first author's name, title, country, year of publication; (2) animal model information: animal strain, gender, age/body weight, number per group, model establishments; (3) administration information: dose, duration, CP preparations (aqueous or ethanol).

Outcome measurements were as follows: blood glucose level (mg/dL), AUC of OGTT (mmol^*^L^−1^^*^h), TC (mg/dL), TG (mg/dL), HDL (mmol/L), LDL (mmol/L) all expressed as mean ± standard deviation (SD). Data presented in graphs were extracted by Web-plot Digitizer software from https://automeris.io/WebPlotDigitizer/. Data from different dosages in one article were treated as separate data reports. Questions regarding the data were managed by sending E-mails to the corresponding authors.

### Assessment of Risk of Bias

The risk of bias and quality of eligible articles was assessed by SYRCLE's risk of bias tool for animal studies (21). The SYRCLE tool is currently the most commonly used assessment tool that is specifically aimed at measuring the risk of bias in animal experiments. It contains 10 items that represent different aspects of risk of bias. Final results of the evaluation are expressed as “low risk,” “high risk,” or “unclear risk.” According to the instructions, the results of assessment should be presented in a table or chart. In the current study, two researchers evaluated the risk of bias independently after training. Disaccords were resolved by discussion.

### Meta-Analysis

Meta-analyses in this article were performed using RevMan 5.3 and STATA 14.0 software. The effect size was expressed as the standardized mean difference (SMD) with a 95% confidence interval (CI). *P* < 0.05 was considered as statistically significant. All data were calculated using a random effects model and using Hedges' method.

#### Assessment and Identification of Heterogeneity

In order to quantify the influence of heterogeneity on meta-analysis, a Higgins' I^2^ test (22) was conducted to examine the heterogeneity between eligible articles. I^2^ ranges from 0 to 100% indicated no heterogeneity up to a high potential of heterogeneity.

To identify the source of possible heterogeneity, subgroup analysis, meta-regression, and sensitivity tests were conducted based on different factors. Each outcome was divided into subgroups according to extract (AE, EE), and dose (<1,000, 1,000–2,000, 4,000, 8,000 mg/d). In addition, meta-regression was performed according to the same factors. Meta-regression detects the correlation of each factor (extract, dose, duration, model generation) to outcomes, *P* < 0.05 indicated high relevance between a studied factor and the outcome. In the meta-regression analyses, dose and duration of treatment were treated as quantitative variables and different animal models and extracts were treated as dummy variables (non-quantitative variables). Furthermore, sensitivity tests were performed by removing those studies with a high risk of bias from the meta-analysis.

#### Assessment of Publication Bias

Since articles with positive results are more likely to be accepted and published, results of meta-analyses might be affected by publication bias. Therefore, a funnel plot was conducted to visually assess the publication bias. The estimation of effect size from each included study was plotted against a measure of precision (standard error) in the funnel plot; asymmetry in a funnel plot indicates the presence of publication bias (23). Additionally, Egger's linear regression test (24) was adopted to assess the publication bias.

Where possible, trim and fill analyses were performed to verify the influence of publication bias on the overall pooled effect size. The trim and fill method was first proposed by Duval and Tweedie (25); it aims to identify and correct asymmetry in funnel plots caused by publication bias. The trim and fill method not only evaluates the number of unpublished papers (missing data) in the meta-analysis, but also fills-in the missed data in the analysis to calibrate the pooled result.

## Results

### Description of Studies

#### Study Selection

**Figure 1:**
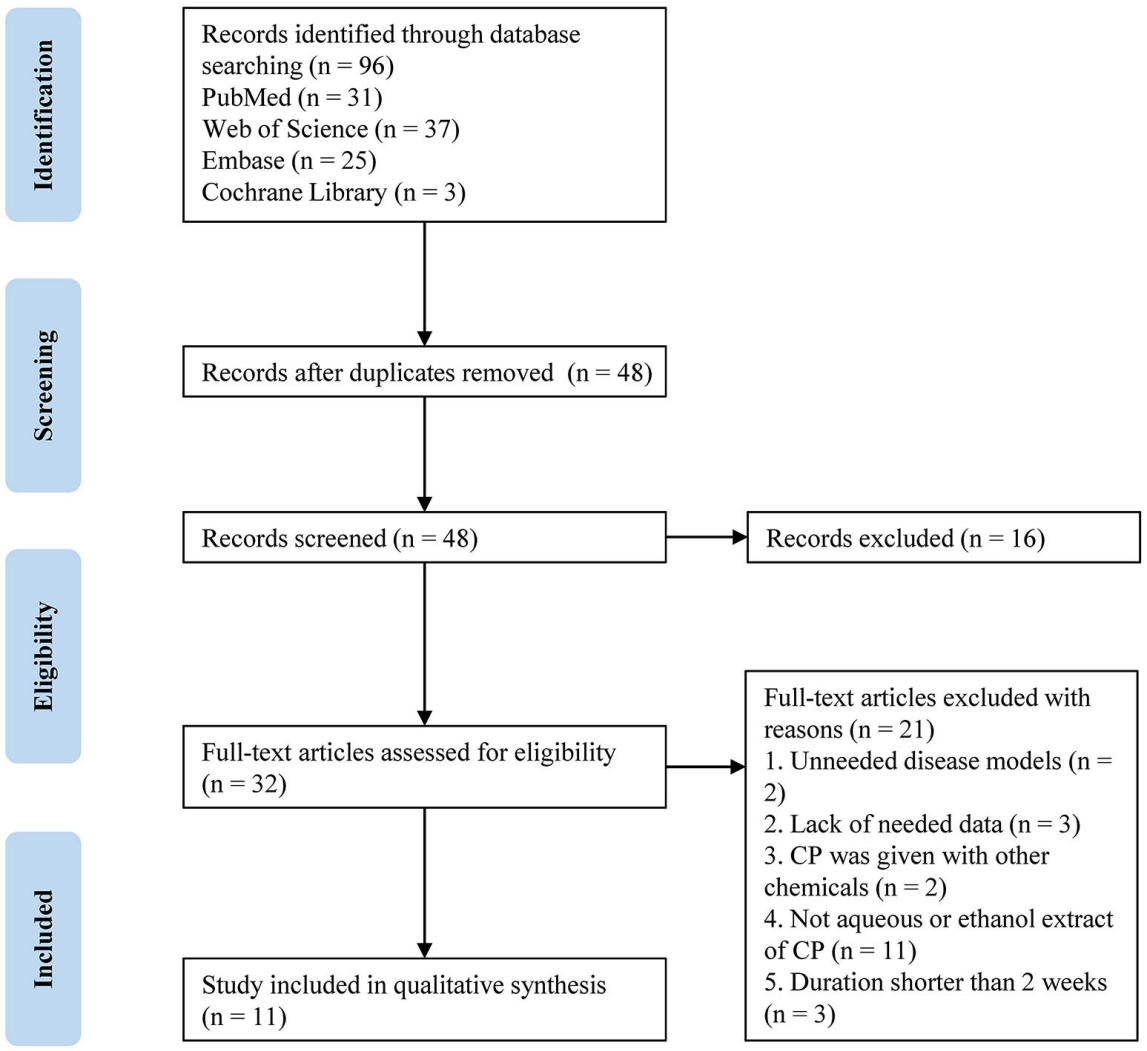
Flow diagram for article selection for the meta-analysis. CP, *Cyclocarya paliurus*.

As presented in the PRISMA flow chart (Figure 1), a total of 96 articles were identified from four databases (PubMed: 31, Web of Science: 37, Embase: 25, and Cochrane Library: 3), out of which 48 duplicates were removed. The remaining articles were viewed by title and abstract according to the inclusion criteria; 16 papers were eliminated for listed reasons and 32 papers were further checked by full-text. Finally, 11 articles [8–11, 15–17, and 26–29] (incorporating 31 data reports) were involved in the meta-analysis and six more articles (18, 26–30) were involved in the systematic review.

#### Characteristics of Included Studies

The basic information of articles included in the meta-analysis is presented in Table 1. Of these articles, nine addressed the use of CPAE and five addressed CPEE. BG data were extracted from nine articles; TC and TG data were acquired from eight articles; HDL and LDL data were acquired from six articles; and OGTT data from four articles. Dosages ranged from 47 to 8,000 mg/kg body weight, and the duration of included studies were all longer than 3 weeks. Animal models used in the studies could be divided into three types: (1) genetic models: KK-A^y^ mice, SHR/cp mice; (2) high-fat-diet (HFD)-induced models: KM mice with HFD; (3) HFD and chemical-induced models: streptozotocin (STZ) and alloxan are two chemicals widely used in developing diabetic models as both can cause damage to pancreatic β cells (35).

**Table 1:** Characteristics of articles involved in the meta-analysis.

**Study**	**Country**	**Extract**	**Animal model**	**Gender**	**Number per group**	**Dose(mg/kg)**	**Duration (weeks)**	**Outcome**
Jiang et al. (31)	China	Ethanol, Aqueous	KM mice + HFD	Male	10	Ethanol 1,500 Aqueous 1,500	10	TC, TG, HDL, LDL
Kurihara et al. (32)	Japan	Aqueous	KK-A^y^	Male	11	2,000	3	BG
Li et al. (33)	China	Aqueous	SD rats + HFD + STZ (35 mg/kg)	Male	6	200	12	BG, TC, TG
Liu et al. (15)	China	Ethanol, Aqueous	C57BL/6 mice + HFD + STZ (40 mg/kg)	Male	6	Ethanol 8,000 (5 groups) Aqueous 8,000 (5 groups)	4	BG, TC, TG, HDL, LDL, OGTT
Ma et al. (11)	China	Ethanol	KM mice + HFD	Male	8	Ethanol 370/750/1,500	4	TC, TG, HDL, LDL
Sheng et al. (17)	China	Aqueous	SD rats + HFD + alloxan (105 mg/kg)	Male	8	Aqueous 1,000	5	BG, TC, TG, HDL, LDL, OGTT
Wang et al. (16)	China	Ethanol, Aqueous	SD rats + HFD + 5% sucrose-containing water + STZ (30 mg/kg)	Male	6	Ethanol 2,000/4,000/8,000 Aqueous 2,000/4,000/8,000	4	BG, TC, TG, HDL, LDL
Wang et al. (34)	China	Aqueous	Wistar rats + HFD + STZ (35 mg/kg)	Male	Model:13 Low dose: 9 High dose: 11	Aqueous 47/94	8	BG
Xiao et al. (9)	China	Aqueous	C57/BL6J mice + HFD + STZ (25 mg/kg)	Male	8	Aqueous 1,000/2,000/4,000	5	BG, TC, TG, HDL, LDL, OGTT
Xu et al. (10)	China	Aqueous	SHR/cp rats	Male	6	Aqueous 500	7	BG, TC, TG
Zhao et al. (8)	China	Ethanol	C57BL/6J mice + HFD + STZ (40 mg/kg)	Male and Female	5	Ethanol 500	4	BG, OGTT

*BG, blood glucose level; HDL, high-density lipoprotein; LDL, low-density lipoprotein; OGTT, oral glucose tolerance test; TC, total cholesterol; TG, triglycerides*.

#### Risk of Bias

**Figure 2:**
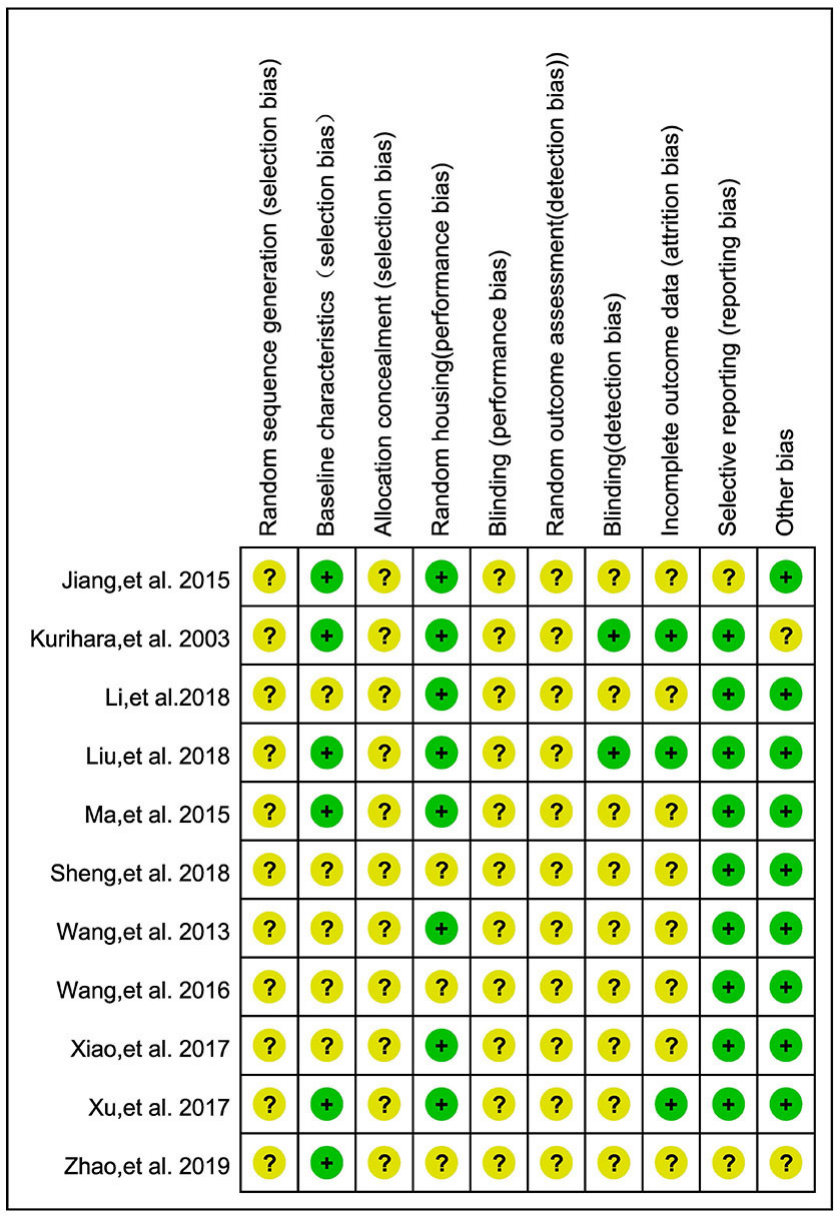
Risk of bias of 11 studies included in the meta-analysis. Yellow dots, unclear risk; green dots, low risk.

A summary of the risk of bias for articles involved in meta-analyses is presented in Figure 2. Eleven articles showed unclear risk in most items using SYRCLE's tool for a lack of report of sequence generation, allocation concealment, and blinding. However, because the results of included outcomes were all objective parameters and difficult to be skewed, the blinding method for outcome assessment may not weigh as much in this meta-analysis when compared with other items.

### Chemicals in CPAE and CPEE

Data of active compounds were acquired from articles involved in the systematic review (Table 2). CPAE mainly contains polysaccharides and CPEE is enriched with flavonoids and triterpenoids, while it contains almost no polysaccharides. In addition, polyphenols can be found in both extracts.

**Table 2:** Constituents detected from aqueous and ethanol extract of *Cyclocarya paliurus*.

**Extract**	**Study**	**Yield**	**Polysaccharides**	**Total flavonoids (mg/g)**	**Total triterpenoids (mg/g)**	**Total phenolic acids (mg/g)**
CPAE	Liu et al. (15)	56.1%	34.5 ±2.02(mg/g)	0.55 ± 0.003	0.08 ± 0.004	0.12 ± 0.003
		53.1%	44.0 ± 5.54(mg/g)	1.99 ± 0.021	0.15 ± 0.002	1.53 ± 0.026
		54.5%	53.6 ± 4.05(mg/g)	2.03 ± 0.001	0.13 ± 0.001	0.91 ± 0.003
		58.0%	28.8 ± 4.91(mg/g)	0.52 ± 0.003	0.05 ± 0.003	0.06 ± 0.003
		53.6%	28.2 ± 3.07(mg/g)	0.60 ± 0.010	0.07 ± 0.001	0.13 ± 0.009
	Wang et al. (16)	21.2%	4.13 ± 0.072(mg/g)	6.75 ± 0.034	—	—
	Wang et al. (34)	4.98%	479.3 ± 19.8(mg/g)	—	—	**Total polyphenols** 38.3 ± 2.3
	Xu et al. (10)	1.5%	68.11%	—	—	—
CPEE	Wang et al. (16)	18.64%	ND	30.41 ± 0.31	—	—
	Liu et al. (15)	48.7%	ND	6.88 ± 0.011	5.69 ± 0.012	0.43 ± 0.014
		50.1%	ND	4.70 ± 0.002	4.39 ± 0.004	1.57 ± 0.154
		49.1%	ND	6.80 ± 0.006	8.15± 0.018	1.09 ± 0.023
		48.1%	ND	5.90 ± 0.008	6.70 ± 0.008	0.71 ± 0.034
		48.5%	ND	2.30 ± 0.001	7.70 ± 0.001	0.47 ± 0.007

*Content values are presented as mean ± SD. CPAE, Cyclocarya paliurus aqueous extract; CPEE, Cyclocarya paliurus ethanol extract; ND, not detected*.

The major flavonoids identified from CP were: (1) quercetin-3-O-glucuronide, (2) quercetin-3-O-galactoside, (3) isoquercitrin, (4) kaempferol-3-O-glucuronide, (5) kaempferol-3-O-glucoside, (6) rhamnoside, (7) kaempferol-3-O-rhamnoside, (8) kaempferol-3-O-α-L-rhamnopyranoside, (9) kaempferol-3-O-β-D-glucuronide, and (10) quercetin (9, 11, 15, 27). Triterpenoids from CP mainly contained: (1) arjunolic acid, (2) cyclocaric acid B, (3) pterocaryoside B, (4) pterocaryoside A, (5) hederagenin, and (6) oleanolic acid (11, 15). Polyphenols from CP were mainly phenolic acids, which included: (1) 3-O-caffeoylquinic acid, (2) 4-O-caffeoylquinic acid, (3) 4,5-di-O-caffeoylquinic acid, (4) 1-caffeoylquinic acid, (5) chlorogenic acid, (6) 5-caffeoylquinic acid, and (7) cryptochlorogenic acid (9, 15, 27). Though involved studies did not analyze the polysaccharides, other articles reported that the polysaccharide portion in CP is composed of: (1) rhamnose, (2) arabinose, (3) xylose, (4) mannose, (5) glucose, and (6) galactose (19, 36, 37).

### Mechanisms Investigated in Previous Studies

Molecular mechanisms from *in vivo* studies investigated in previous articles are summarized in Table 3. Only parameters showing statistical differences are presented.

**Table 3:** Mechanisms investigated in animal studies from 17 articles involved in the systematic review.

**Study**	**Disease model**	**Pharmaceutical composition**	**Animal**	**Gender**	**Pathways**	**Protein/gene detected**	**Major findings**
Jiang et al. (31)	Hyperlipidemia	CPAE CPEE	KM mice	M	—	HMG-CoA reductase↓(EE), CYP7A1↑(EE), ACA2↓(EE)	BW↓, food intake↓, TC↓(EE), TG↓(EE), AST↓(EE), ALT↓(EE), CRE↓(EE), BUN↓(EE), UA↓(EE), hepatic TC↓(EE), TG↓(EE), adipose size↓(EE), heart weight↓(AE), fat weight↓(EE), hepatic bile acid↑(EE), fecal bile acid↑(EE)
Jiang et al. (7)	Adipose dysfunction and insulin resistance	CPEE	KM mice	M	Insulin-Signaling: IRS-1, AKT	TNF-α↓, IL-6↓, MCP-1↓, resistin↓, adiponecin↓, p-Akt/Akt↑, Ser307/IRS-1↓, PY99/IRS-1↑	Hepatic and muscle glycogen↑, AUC of OGTT↓
Kurihara et al. (32)	Type 2 diabetes	CPAE	KK-A^y^ mice	M	—	—	AUC of OGTT↓, BG↓, α-glucosidase activity↓
Kurihara et al. (30)	Post-prandial hyperlipemia	CPAE	ICR mice	M	—	—	TG↓, pancreatic lipase↓
Li et al. (29)	Type 2 diabetes	CPAE	KM mice	—	—	—	Percentage of glycemic↓
Li et al. (33)	Diabetes	CPAE	SD Rats	M	Cell proliferation, cellular response to glucose, insulin stimulus	Ddit4↑, Fgf21↑, Ins1↑, Ins2↑, miR-200↓, miR-375↓, Aldh1b1↑, Hps5↑	FBG↓, AUC of OGTT↓, SI↑, islets area/total pancreatic area↑, thickness of glomerular basement membrane↓, TC↓, TG↓, HDL-c/LDL-c↑, AST↓, ALT↓, CRE↓, BUN↓, FFA↓, SOD↑, MDA↓, glutathione↑, TNF-α↓, IL-6↓, Ki67 positive cells↑
Liu et al. (15)	Type 2 diabetes	CPEE CPAE	C57BL/6	M	—	—	FBG↓, BW↑, AUC of OGTT↓, AUC of ITT↓, TC↓, TG↓, HDL-C↑(EE), LDL-C↓(EE), AST↓, ALT↓, TBIL↓, CRE↓, BUN↓
Ma et al. (11)	Hyperlipidemic	CPEE	KM mice	M	TNF-α/p38MAPK pathways: total- and TRL-apoB48, TNF-α, p38MAPK	Total- and TRL-apoB48↓, TNF-α↓, p-p38MAPK↓ (intestinal tissue)	BW↓, food intake↓, TC↓, TG↓, HDL-C↑, LDL-C↓, AST↓, ALT↓, CRE↓, BUN↓, MDA↓, SOD↑, GSH-Px↑
Sheng et al. (17)	Type 2 diabetes	CPAE	SD rats	M	—	—	FBG↓, AST↓, ALT↓, BUN↓
Wang et al. (16)	Type 2 diabetes	CPEE CPAE	SD rats	M	—	—	Food intake↓, BG↓, FINS↓, GSH-Px↑, FFA↓, TC↓, TG↓, LDL–C↓, HDL–C↑, SOD↑, MDA↓, BUN↓, CRE↓(EE), GSP↓
Wang et al. (34)	Diabetic nephropathy	CPAE	Wistar albino rats	M	—	—	BG↓, SI↑, CRE↓, BUN↓, Upro↓, UG↓, UCr↓, CysC↓, α1-MG levels↓, RI↓, IL-6↓, ET-1↓, T-AOC↑, SOD↑, MDA↓, GSH-Px↓, CAT↑, renal AR activity↓
Xiao et al. (9)	Type 2 diabetes	CPAE	C57/BL6J mice	M	MAPK pathway: p38, ERK, JNK; Akt pathway	Bax/Bcl-2↓, caspase-8↓, Caspase-9↓, cleaved caspase-3↓, p-p38 MAPK↓, p-ERK↓, p-JNK↓, p-Akt↑	BW↑, BG↓, SI↑, AUC of OGTT↓, AST↓, ALT↓, ALP↓, TC↓, TG↓, HDL↑, LDL↓, CRE↓
Xu et al. (10)	Metabolic syndrome	CPAE	SHR/cp rats	M	Insulin-Signaling Pathway: IRS1, InsR, PI3K, AKT, FoxO1, POMC, NPY	p-InsR↑, p-IRS1tyr989↑, m-PI3Kp85/T-PI3Kp85↑, p-Akt↑, p-FoxO1↑, POMC↑, NPY↓	Food intake↓, BW↓, epididymal fat mass↓, abdominal fat mass↓, FBG↓, FINS↓, TC↓, TG↓, FFA↓, GOT↓, GPT↓, T-GSH↑, SOD↑, MDA↓
Yao et al. (28)	Hyperlipidemia and obesity	CPEE	SD rats	M	—	—	BW↓, TC↓, TG↓, HDL-C↑, LDL-C↓, AST↓, ALT↓, CRE↓, BUN↓, UA↓, fat mass↓, FFA↓, serum leptin↓, serum adiponectin↑, hepatic TG↓, hepatic TC↓, fecal and hepatic bile acid↑, serum total apoB48↓
Yoshitomi et al. (27)	Type 2 diabetes	CPEE	ICR mice	M	—	p-AS160↑, p-AKT↑	BG↓, SI↑
Zhai et al. (26)	Type 2 diabetes	CPEE	C57/BL6J mice		—	—	BW↑, BG↓, serum: TC↓, TG↓, LDL↓, HDL↑, AST↓, ALT↓, hepatic TC↓, hepatic TG↓
Zhao et al. (8)	Type 2 diabetes	CPEE	C57/BL6J mice	F/M	—	—	BW↑, FBG↓, AUC of OGTT↓, SI↓, MDA↓, SOD↑, GSH-Px↑

*AST, aspartate transaminase; ALT, alanine aminotransferase; AR activity, aldose reductase activity; ACA2, acyl-CoA cholesterol acyltransferase 2; apoB48, apolipoproteinB48; ALP, alkaline phosphatase; BAX, Bcl-2 Associated X Protein; Bcl-2, B-cell lymphoma-2; BG, blood/serum glucose; BUN, blood urea nitrogen; BW, Body weight; CAT, catalase; CPAE, Cyclocarya paliurus aqueous extract; CPEE, Cyclocarya paliurus ethanol extract; CRE, creatinine; CYP7A1, cholesterol 7α-hydroxylase; CysC, cystatin C; Ddit4, DNA-damage-inducible transcript 4; ERK, extracellular regulated protein kinases; ET-1, endothelin-1; FBG, fasting blood glucose; FFA, free fatty acids; Fgf21, fibroblast growth factor 21; FINS, fasting insulin; FoxO1, forehead box-containing protein of the O subfamily 1; GOT, glutamic oxaloacetic transaminase; GPT, glutamic pyruvic transaminase; GSH-Px, glutathione peroxidase; GSP, glycated serum protein; HDL-C, high-density lipoprotein cholesterol; HMG-CoA, 3-hydroxy-3-methylglutaryl-coenzyme A; JNK, c-Jun N-terminal kinase; IL-6, interleukin 6; IRS-1, insulin receptor substrate; ITT, insulin tolerance test; LDL-C, low-density lipoprotein cholesterol; MAPK, mitogen-activated protein kinase; MCP-1, monocyte chemotactic protein 1; MDA, malondialdehyde; NPY, neuropeptide Y; OGTT, oral glucose tolerance test; PI3K, phosphatidylinositol 3-kinase; POMC, proopiomelanocortin; RI, renal index; SI, serum insulin; SOD, superoxide dismutase; T-AOC, total antioxidant capability; T-GSH, total-glutathione; TRL-apoB48, triglyceride-rich apoB48; TC, total cholesterol; TG, triglycerides; TNF-α, tumor necrosis factor-α; TBIL, total bilirubin; UA, uric acid; UCr, urine creatinine; UG, urine glucose; Upro, 24 h urine protein; α1-MG, α1 microglobulin. ↑: up regulated*.

*↓: down regulated*.

### Results From Meta-Analyses

#### Effect of CP on Glucose Metabolism

**Figure 3:**
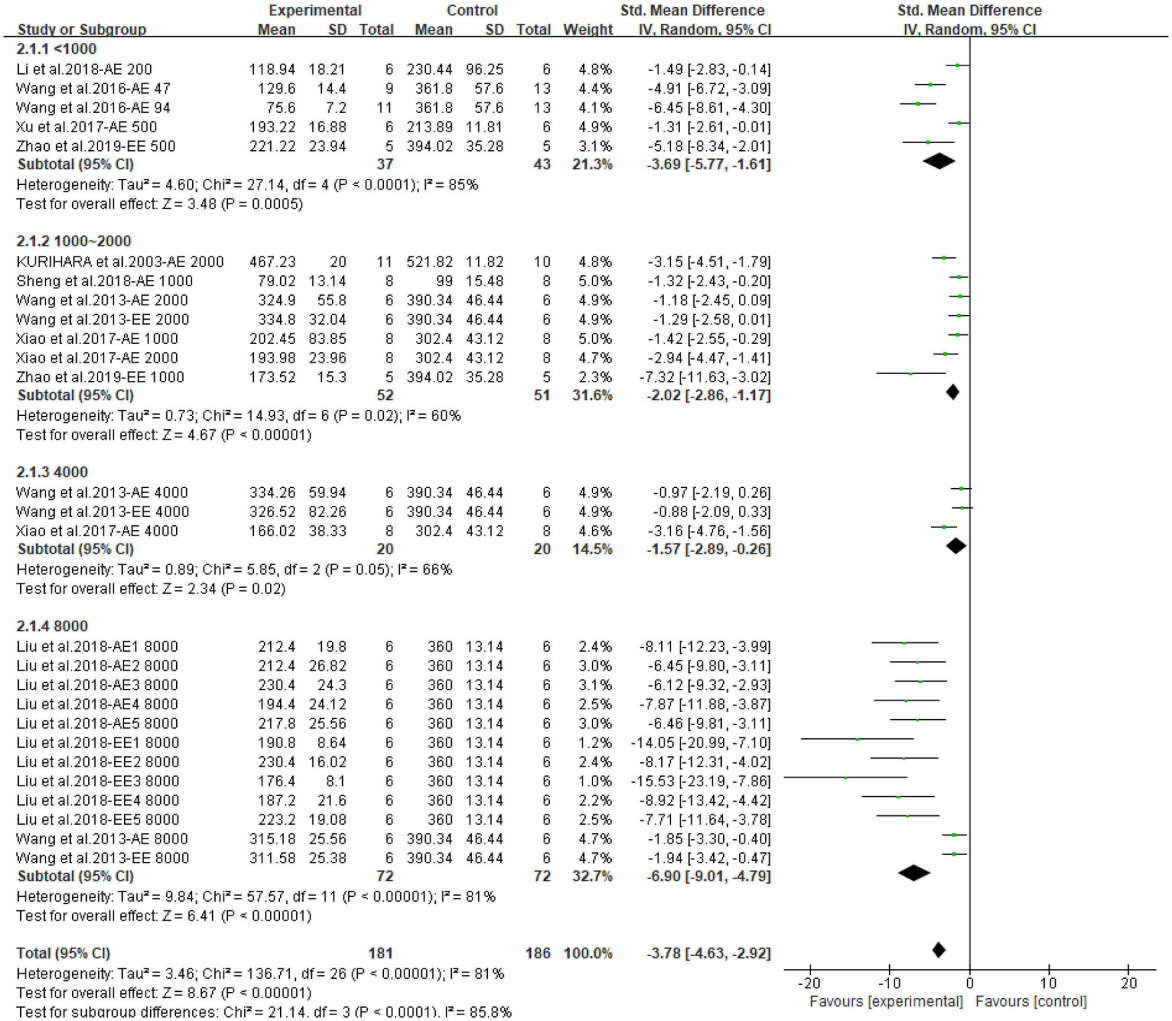
Forest plot for the effect of *Cyclocarya paliurus* (CP) on blood glucose levels in animal models. Subgroup analysis was performed according to doses.

**Figure 4:**
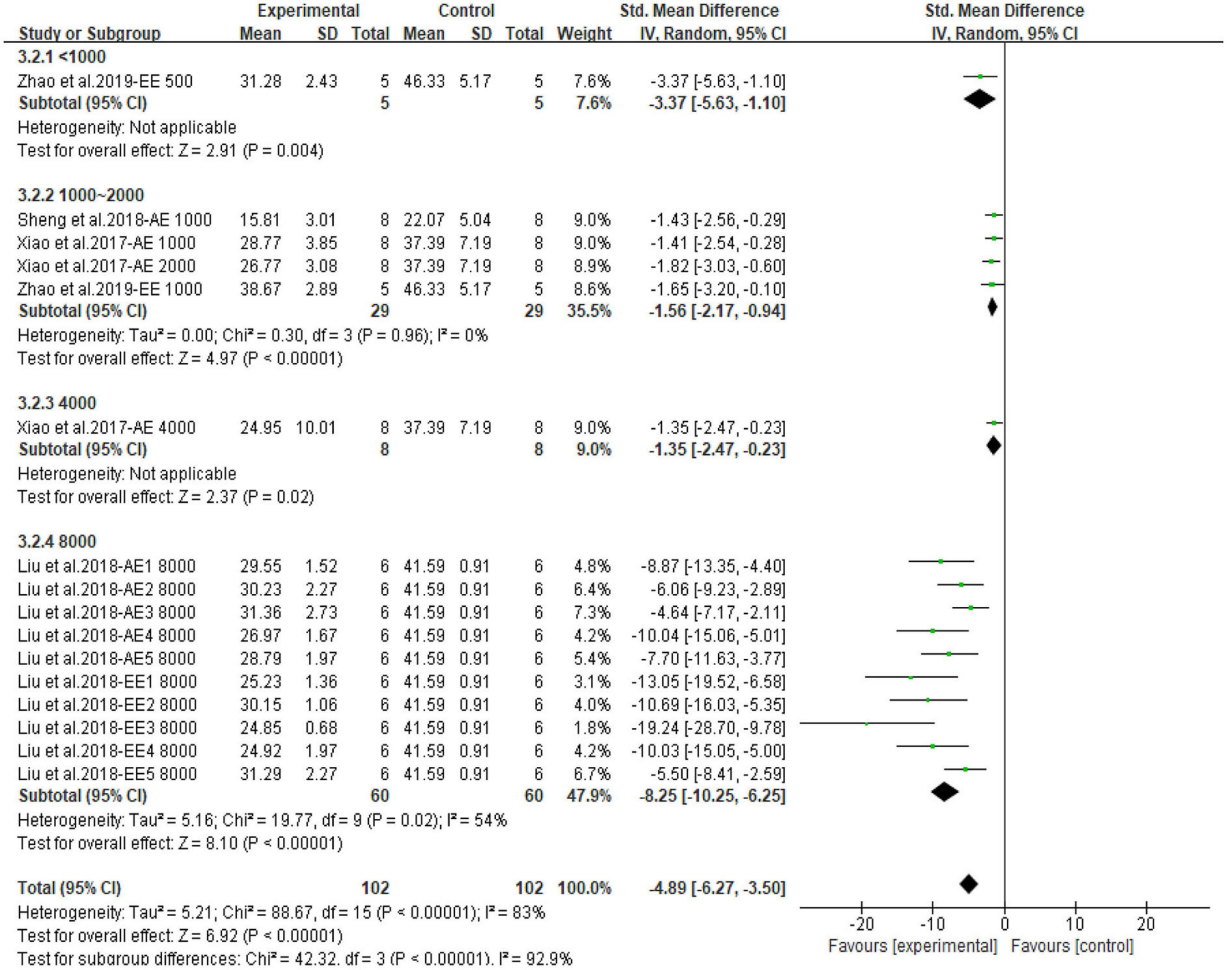
Forest plot for the effect of *Cyclocarya paliurus* (CP) on area under curve of oral glucose tolerance tests in animal models. Subgroup analysis was performed according to doses.

Both CPAE and CPEE showed definite hypoglycemic effects in animal models. CP extracts could lower BG level with an SMD of −3.20 (95% CI: −4.09, −2.31; *P* < 0.001) and −5.73 (95% CI: −7.90, −3.56; *P* < 0.001). Heterogeneity in the two subgroups was high (I^2^ = 79, 85%, respectively) (Supplementary Figure 1). Meta-regression showed that the effect size of CP on BG was related to the dosage used in treatment (*P* < 0.05), but not related to duration, model generation, or extract types. For the AUC of OGTT, the SMD results were −3.64 (95% CI: −5.06, −2.23; *P* < 0.001) for CPAE and −7.62 (95% CI: −11.01, −4.23) for CPEE (Supplementary Figure 2). Similarly, meta-regression showed the effect size was proportional to the dosage used in treatment. Furthermore, heterogeneity in subgroup analysis according to dosage (<1,000, 1,000–2,000, 4,000, 8,000 mg/d) was partially reduced (Figures 3, 4). Sensitivity analyses were carried out by eliminating data from studies with a high risk of bias, however, they did not significantly reduce the heterogeneity between studies (Supplementary Table 2).

#### Effect of CP on Lipid Metabolism

**Figure 5:**
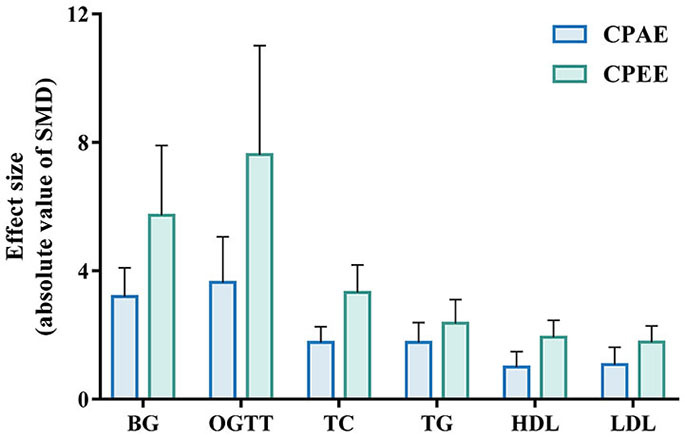
Effect size of CPAE and CPEE on included parameters. CPAE, *Cyclocarya paliurus* aqueous extract; CPEE, *Cyclocarya paliurus* ethanol extract; BG, blood glucose level; OGTT, oral glucose tolerance test; TC, total cholesterol; TG, triglyceride; HDL, high-density lipoprotein; LDL, low-density lipoprotein.

**Figure 6:**
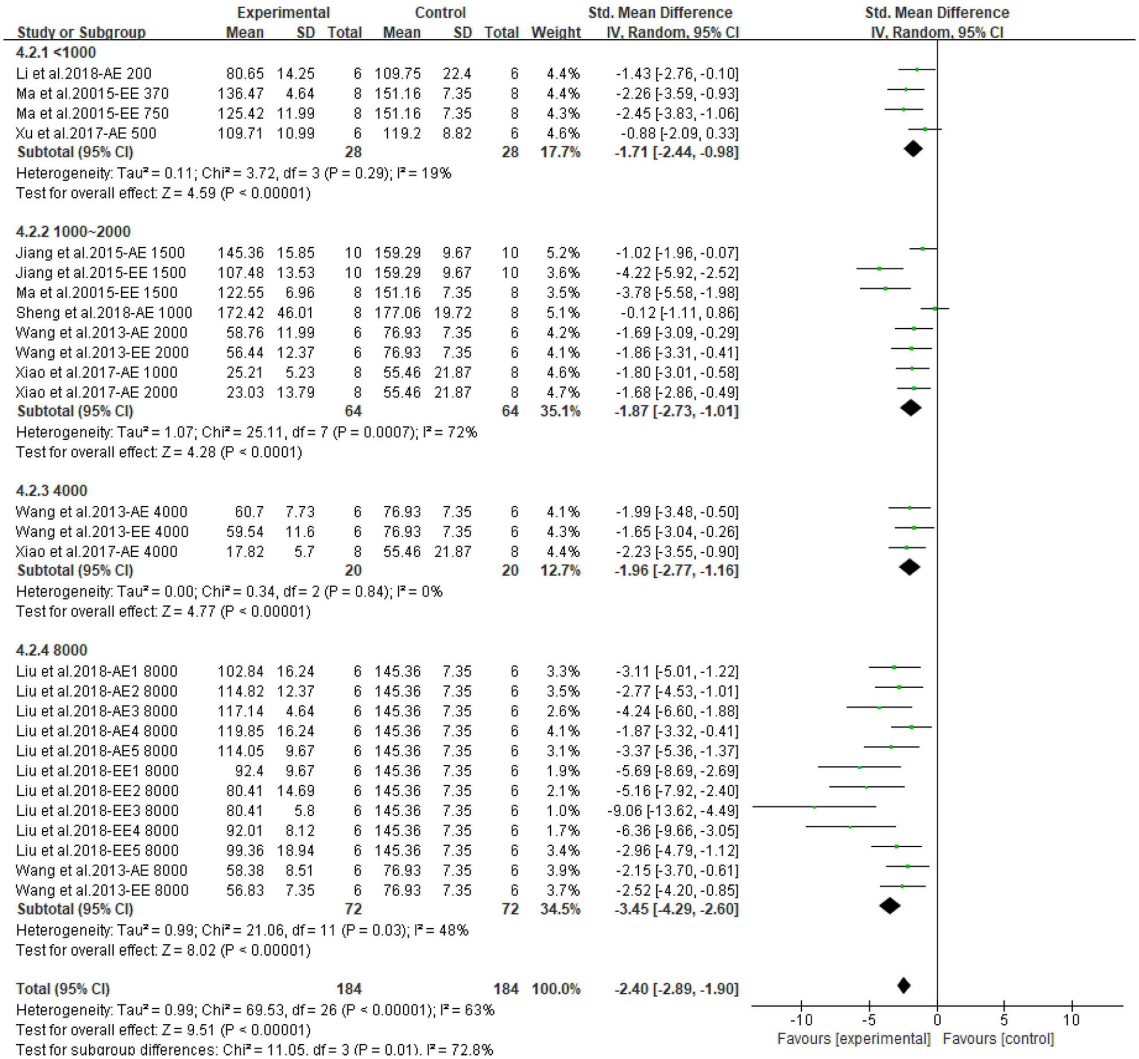
Forest plot for the effect of *Cyclocarya paliurus* (CP) on total cholesterol levels in animal models. Subgroup analysis was performed according to doses.

**Figure 7:**
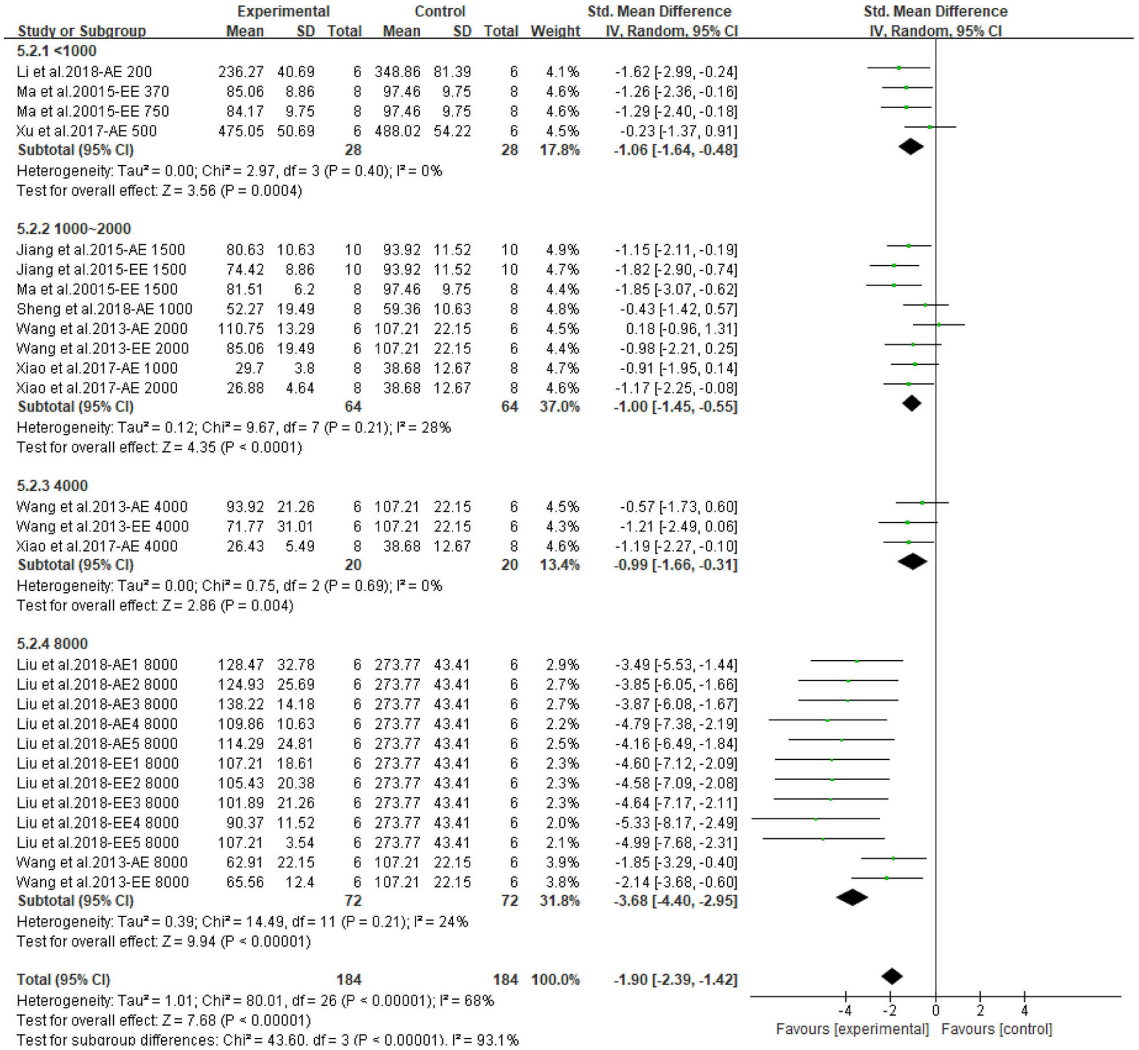
Forest plot for the effect of *Cyclocarya paliurus* (CP) on triglyceride levels in animal models. Subgroup analysis was performed according to doses.

**Figure 8:**
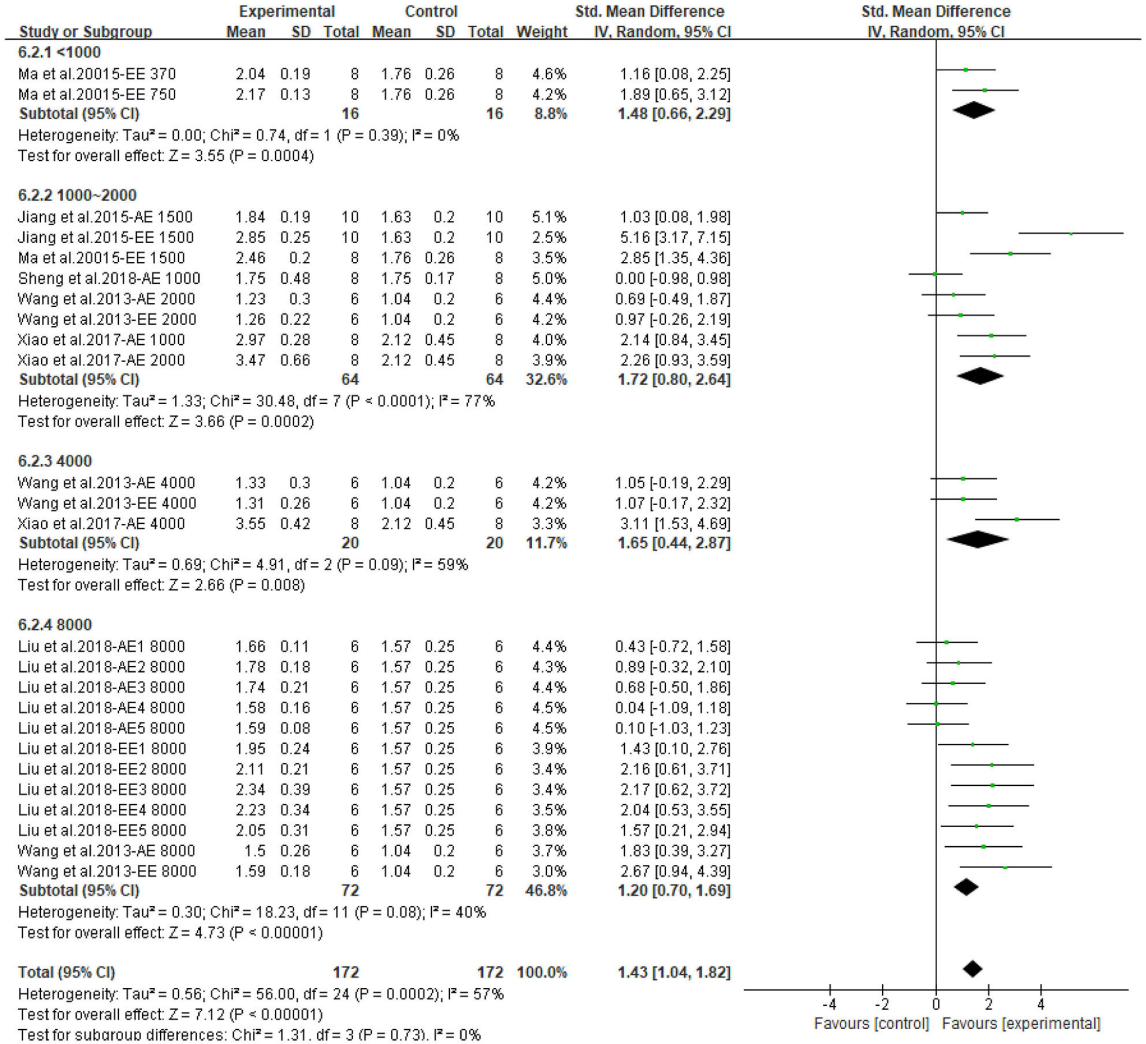
Forest plot for the effect of *Cyclocarya paliurus* (CP) on high-density lipoprotein levels in animal models. Subgroup analysis was performed according to doses.

**Figure 9:**
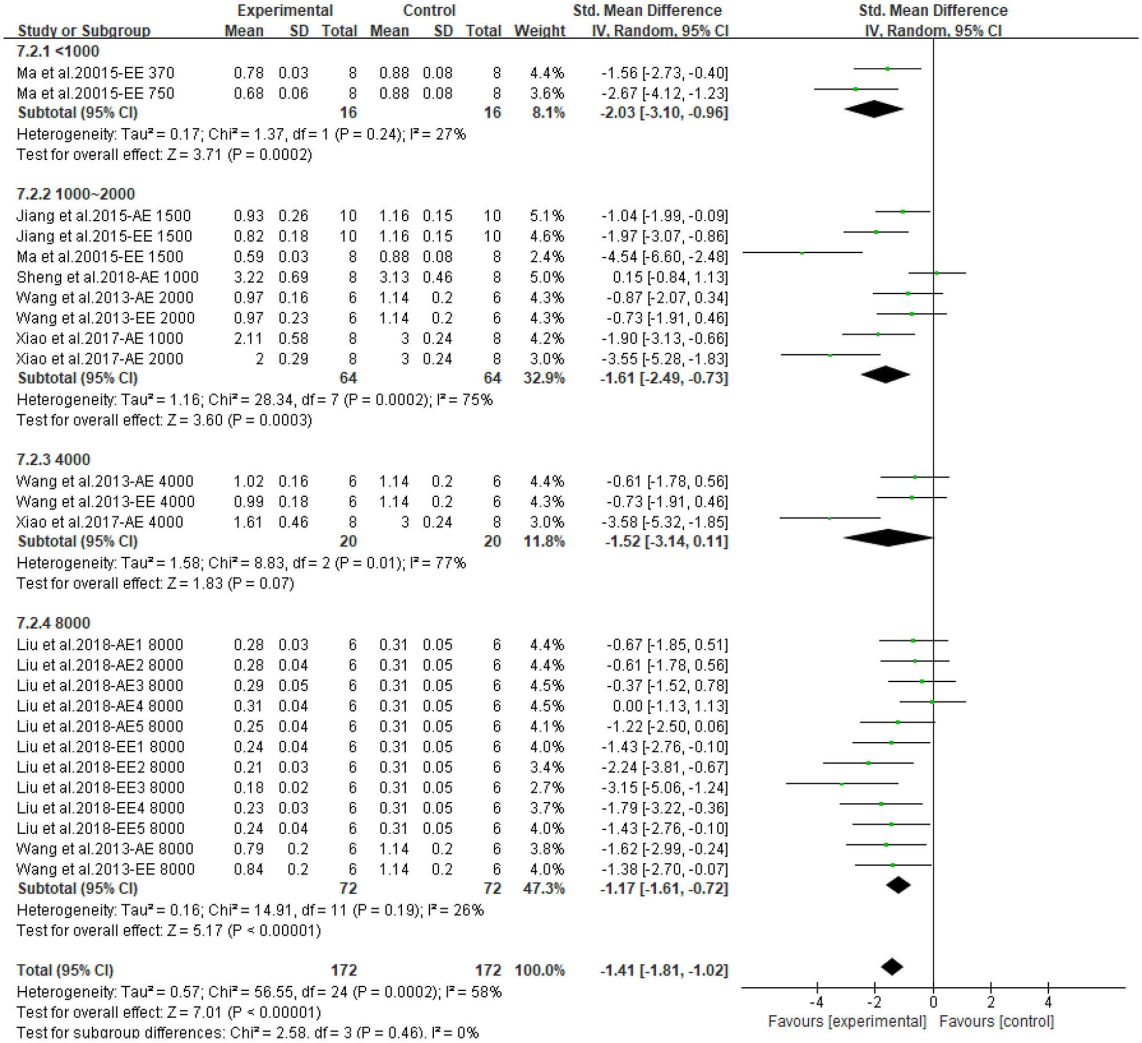
Forest plot for the effect of *Cyclocarya paliurus* (CP) on low-density lipoprotein levels in animal models. Subgroup analysis was performed according to doses.

The meta-analysis showed that CP has a beneficial effect on lipid profile. All outcomes could be improved by CPAE and CPEE. The overall pooled effect for TC, TG, HDL, LDL were −2.40 (95% CI: −2.89, −1.90; *P* < 0.001), −1.90 (95% CI: −2.39, −1.42; *P* < 0.001), 1.43 (95% CI: 1.04, 1.82; *P* < 0.001), and −1.41 (95% CI: −1.81, −1.02; *P* < 0.001), respectively. Forest plots for subgroup meta-analysis according to different extracts are shown in Supplementary Figures 3–6. In addition, Figure 5 compares the effect size of CPAE and CPEE on each parameter. Figures 6–9 are the forest plots generated according to different doses as subgroups.

#### Publication Bias

**Figure 10:**
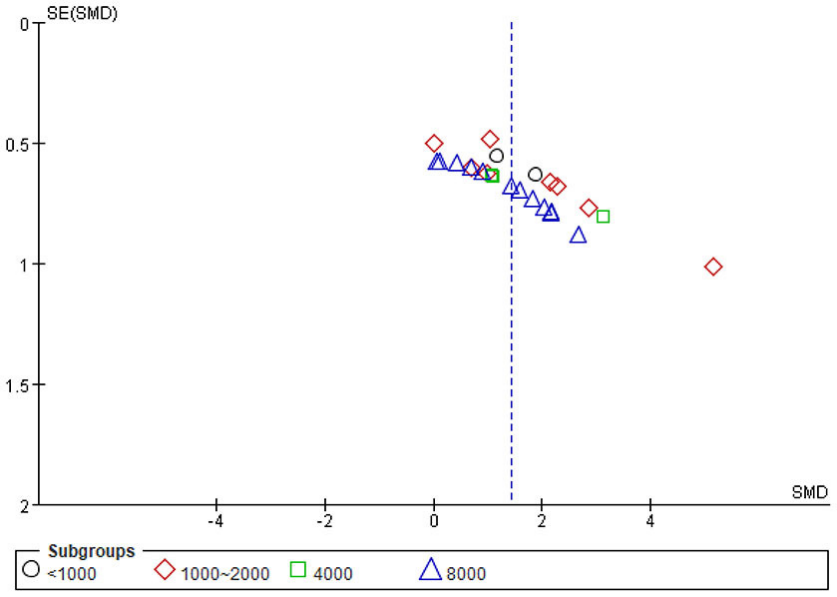
Funnel plot for publication bias in the meta-analysis.

Asymmetry in the funnel plots indicates potential publication bias in the meta-analysis (Figure 10), the result was further confirmed by Egger's test with a *P*-value < 0.01. Sample size in studies involved in this meta-analysis were rather small (no larger than 13), which could have caused an over-estimation of the overall effect size (38).

Only the result on HDL levels could be remedied by the trim and fill method (Supplementary Figure 7). Although the effect size on HDL level was estimated to be smaller, with an SMD = 0.936 (95% CI: 0.498, 1.375; *P* < 0.001), the beneficial effect was still steady. The trim and fill method failed to perform on other outcomes, possibly because of the existence of other biases or extremely positive and highly-weighted data (39).

## Discussion

Though CP has been used as a health-enhancing herbal tea for hundreds of years, the active components and underlying mechanisms still need to be uncovered. Various studies report CP's effect on regulating glucose homeostasis and improving lipid profiles, but no evidence-based research has been performed regarding its bioactivity for regulating glucolipid metabolism. This article aimed to collect current evidence to investigate the effect of CP on glucolipid metabolism through a meta-analysis. BG level and OGTT results were used to measure the hypoglycemic effect; TC, TG, HDL, and LDL were used to assess its effect on hyperlipidemia. The results showed that in animal studies, oral intake of CP extracts could effectively benefit glucolipid metabolism. BG level, as well as the AUC of OGTT were decreased; TC, TG, and LDL levels were decreased, while HDL level was increased. The effects of CP on glucolipid metabolism showed a dose-dependent manner: as the dosage rose, the effect size of the subgroup became larger.

Metabolic syndrome is a systematic metabolic dysfunction that involves insulin resistance, visceral adiposity, dyslipidemia, and hypertension (40). Insulin resistance is known to be the primary mechanism (41, 42), while other underlying mechanisms including oxidative stress (43), inflammation (44, 45), and endothelial dysfunction (46) are also discussed in literature. As is indicated by the criteria for clinical diagnosis of metabolic syndrome, abnormal carbohydrate and lipid metabolism are highly associated with this syndrome (5).

After its secretion by the pancreatic β cells, insulin binds with insulin receptors on target cells to perform its biofunctions. Obstruction to insulin signaling pathways could lead to insulin resistance (47), causing hyperinsulinemia, which encourages other pathological changes associated with metabolic syndrome, including dyslipidemia, hypertension, as well as the progress of type 2 diabetes (42). CP was shown to decrease food intake in mice (10, 11, 31), this effect is probably due to its function in improving insulin signaling in the hypothalamus, and thus regulating the appetite regulating neuron neuropeptide Y (NPY) and proopiomelanocortin (POMC), leading to inhibition of food intake (10). In muscle tissues, CP also increases the activation of insulin receptor substrate-1(IRS-1), which further activates downstream proteins, such as phosphoinositide 3-kinases (PI3K), protein kinase B (Akt), and glucose transporter type 4 (GLUT4), and enhances glucose disposal in muscles (7).

It has been suggested that insulin resistance and hyperinsulinemia would then cause increasing pancreatic β cell apoptosis, leading to a decrease in β cell mass, as a loss of β cell mass due to apoptotic death is observed in patients with type 2 diabetes (48, 49). In animal studies, CP was shown to decrease pancreatic β cell apoptosis, possibly due to the regulation of mitogen-activated protein kinase (MAPK) pathways and Akt pathways (9). Furthermore, restoration of β cell mass and function were found in histological observations, moreover, this β-cell-protecting effect was also confirmed by *in vitro* studies in NIT-1 cells (8, 9), which are a pancreatic β cell line established from mice (50).

Oxidative stress and inflammation are also linked to metabolic syndrome. The expression of markers for inflammation and oxidative stress were found to be much higher in patients with metabolic syndrome than those without it (51). Oxidative stress is characterized by the production and degradation of reactive oxygen species (ROS) and over-production of oxidative free radicals (52), which occurs along with inflammation when adipocytes fail to store excessive energy (53). Inflammatory cytokines like tumor necrosis factor-α (TNF-α) down-regulate the activation of insulin receptors (54), and eventually lead to insulin resistance, causing hypertriglyceridemia, low HDL levels and high LDL levels (55). In many current animal studies, CP administration successfully decreases the expression of TNF-α, interleukin-6 (IL-6), malondialdehyde (MDA), and increases superoxide dismutase (SOD) and glutathione peroxidase (GSH-Px) levels, showing its capacity for controlling inflammation and oxidative stress (8, 10, 11, 16).

Other underlying mechanisms have also been investigated in animal models. Apolipoprotein B48 (apoB48) is a protein that transports exogenous fat into the circulation and other body parts (56). ApoB48 promotes the absorption of exogenous lipid in the intestine, and overexpression of apoB48 may result in hyperlipidemia (57). CP significantly lowered serum apoB48 content in hyperlipidemic models, probably because of modulation in the MAPK pathway (11, 28). Leptin and adiponectin are two major adipocytokines, both play important roles in metabolic homeostasis. It has been pointed out that the ratio of leptin to adiponectin is a better indicator of insulin resistance and atherosclerosis than each alone (58). The research of Yao et al. found that CP significantly reduced the leptin to adiponectin ratio, indicating that CP protects against obesity through regulating the secretion of adipocytokines (28). CP was also demonstrated to have the ability to inhibit α-glucosidase activity (30). Furthermore, a transcriptome study revealed more possible mechanisms: CP improved β-cell survival and insulin secretion, and regulated the miR-200/375-Aldh1b1/Hps5-Hes1 co-regulatory network to restore lipid metabolism and exert its anti-oxidant ability (33).

**Figure 11:**
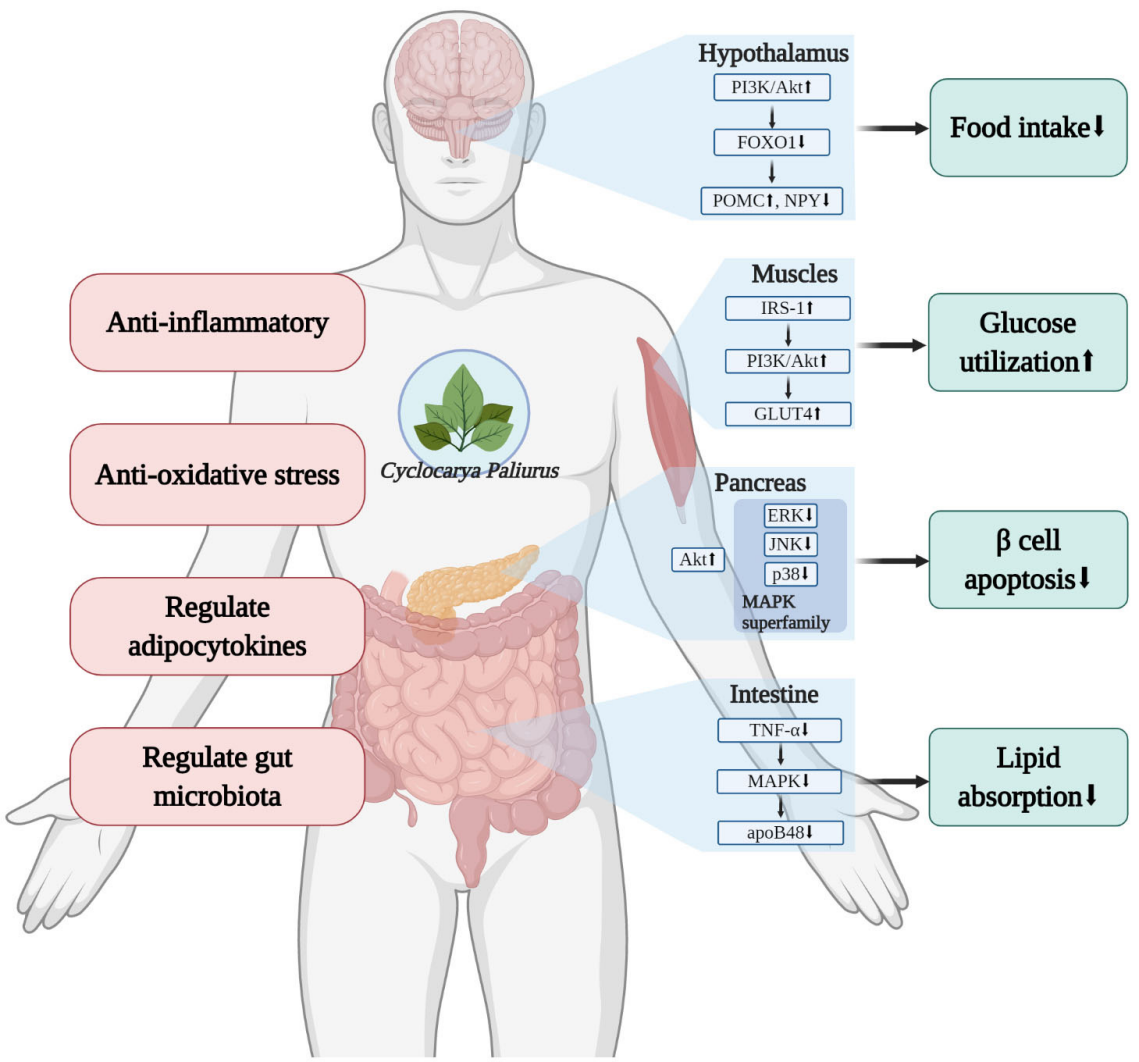
Diagram of the mechanisms investigated for the effect of *Cyclocarya paliurus* (CP) on glucolipid metabolism (created with BioRender.com).

The mechanisms investigated in articles involved in this systematic review are summarized and presented in Figure 11. From the hypothalamus to the intestine, CP targets multiple organs and tissues, and systematically regulates glucolipid metabolism and benefits metabolic syndrome *in vivo*.

It is worth noting that in this meta-analysis, CPEE had a greater effect on every calculated parameter than CPAE (Figure 5). In addition, in some studies involving both extracts, CPEE also seemed to be more effective for improving phenotypes and regulating proteins (15, 31). However, this effect might have been caused by an inherent bias of individual studies or across studies. Furthermore, publication bias and selective reporting may also have affected the cumulative evidence. Flavonoids and triterpenoids are more likely to be the effective compounds in CPEE, and further studies performed on these phytochemicals have shown positive results (13, 18, 59–61). Meanwhile, as the main components found in CPAE, polysaccharides have been previously investigated. For example, polysaccharides in CP could ameliorate hyperlipidemia via regulating DNA methylation levels and regulating lipid metabolism-related enzymes in the liver (36, 37). It has been pointed out that the bioavailability of flavonoids is low, as a large proportion of them will remain in the digestive system after oral intake (62, 63). However, Cheng's study showed that CP flavonoids can modulate gut microbiota to regulate certain metabolic processes, to the benefit of murine obesity models (60).

In addition, a toxicity study reported that, even when given at the highest dosage (10 g/kg in Kunming mice and 20 g/kg in SD rats), CPAE did not result in any mutagenicity, sperm deformity, or teratogenicity in rodents, even when the dose was 150-fold of the recommended human dose (64). This suggests that CP is safe to be used as an additive ingredient in daily nutritional supplements, with 6 g of crude extract proposed to be the effective daily dosage for a 50 kg human (28). No reports regarding any side effects of the oral intake of CP were found, and this point is worth further investigation.

This article has some limitations. Firstly, according to the assessment of risk of bias, the involved studies showed an unclear risk in most items, which is common in animal studies (65). Ambiguous descriptions of experimental methods may have led to skewed outcomes and eventually affected the pooled results of the meta-analysis. Moreover, publication bias may have enlarged the effect size of CP. However, for the inevitable heterogeneity in preclinical studies, the results of this meta-analysis should be interpreted as a guide to the direction of outcomes instead of viewed as precise estimates of the pooled effect size. No random-controlled clinical trials were found in databases, and additional studies are still needed to identify more potential therapeutic targets, and form a more comprehensive understanding of CP.

## Conclusion

According to this meta-analysis, oral intake of CP showed absolute ameliorative effects on glucolipid metabolism in animal models. In addition, the studied molecular pathways in previous literature were systematically reviewed. CP ameliorates metabolic syndrome probably through enhancing insulin sensitivity, decreasing pancreatic β cell apoptosis, inhibiting lipid absorption, regulating adipocytokines and gut microbiota, and combatting inflammation and oxidative stress.

As a daily dietary supplement, CP showed promising potential for dealing with metabolic syndrome by influencing multiple related mechanisms. In addition, future research should focus on the composition of CPEE.

## Data Availability Statement

The original contributions generated for the study are included in the article/Supplementary Material, further inquiries can be directed to the corresponding author/s.

## Author Contributions

YW, WL, TL, and LW designed this research. WL, YH, and SQ participated in the literature search and selection. XG and MW worked on data extraction. YW and WL organized the data and performed the analysis. YW wrote the original manuscript. All authors contributed to the article and approved the submitted version.

## Conflict of Interest

The authors declare that the research was conducted in the absence of any commercial or financial relationships that could be construed as a potential conflict of interest.
