# Supplementary material for: Effects of Cyclocarya paliurus Aqueous and Ethanol Extracts on Glucolipid Metabolism and the Underlying Mechanisms: A Meta-Analysis and Systematic Review
Source: Front Nutr. 2020 Dec 1;7:605605. doi: 10.3389/fnut.2020.605605 (PMC7736088; doi:10.3389/fnut.2020.605605)
Supplement: Supplementary file 1 [file Table_2.DOCX]

| Database | Step | Query | Results |
| --- | --- | --- | --- |
| PubMed | 6 | ((#3) AND (#4)) AND (#5) | 31 |
|  | 5 | (#1) OR (#2) | 1,739,591 |
|  | 4 | (((((((mice[Title/Abstract]) OR (mouse[Title/Abstract])) OR (rat[Title/Abstract])) OR (rats[Title/Abstract])) OR (animal[Title/Abstract])) OR (animals[Title/Abstract])) OR (rodent[Title/Abstract])) OR (rodents[Title/Abstract]) | 3,212,849 |
|  | 3 | (Cyclocarya[Title/Abstract]) OR (&quot;sweet tea tree&quot;[Title/Abstract]) | 107 |
|  | 2 | (((((((diabet*[Title/Abstract]) OR (obes*[Title/Abstract])) OR (hyperlipidemi*[Title/Abstract])) OR (hyperlipemi*[Title/Abstract])) OR (lipidemi*[Title/Abstract])) OR (insulin[Title/Abstract])) OR (glucose[Title/Abstract])) OR (metabolic[Title/Abstract]) | 1,624,017 |
|  | 1 | ((((diabetes mellitus[MeSH Terms]) OR (obesity[MeSH Terms])) OR (hyperlipidemias[MeSH Terms])) OR (insulin resistance[MeSH Terms])) OR (glucose intolerance[MeSH Terms]) | 693,594 |
| Web of Science | 5 | #4 AND#3 | 37 |
|  | 4 | #2 AND #1 | 59 |
|  | 3 | TOPIC: (mice)OR TOPIC: (mouse)OR TOPIC: (rats)OR TOPIC: (rat)OR TOPIC: (rodents)OR TOPIC: (rodent)OR TOPIC: (animals)OR TOPIC: (animal) | 3862920 |
|  | 2 | TOPIC: (diabet*)OR TOPIC: (obes*)OR TOPIC: (diabetes mellitus)OR TOPIC: (obesity)OR TOPIC: (hyperlipidemias)OR TOPIC: (hyperlipidemi*)OR TOPIC: (hyperlipemi*)OR TOPIC: (lipidemi*)OR TOPIC: (insulin)OR TOPIC: (glucose)OR TOPIC: (metabolic) | 1891901 |
|  | 1 | TOPIC: (Cyclocarya) | 188 |
| Embase | 3 | #1AND #2 | 25 |
|  | 2 | Animal:ti,ab,kw OR rat:ti,ab,kw OR mice:ti,ab,kw OR rodent:ti,ab,kw | 2719699 |
|  | 1 | Cyclocarya: ti,ab,kw | 123 |
| Cochrane Library |  | Title Abstract Keyword: Cyclocarya paliurus | 3 |

**Supplementary table 1:** Search terms used in databases.

| **Outcome** | **Subgroup/total** | **SMD (95%CI) before sensitivity test** | **I^2^ before sensitivity test** | **SMD (95%CI) after sensitivity test** | **I^2^ after sensitivity test** |
| --- | --- | --- | --- | --- | --- |
| **BG** | <1000 mg/d | -3.69 (-5.77, -1.61) | 85% | -1.39 (-2.33, -0.46) | 0% |
|  | 1000~2000 mg/d | -2.02 (-2.86, -1.17) | 60% | -1.93 (-2.75, -1.11) | 49% |
|  | 4000 mg/d | -1.57 (-2.89, -0.26) | 66% | -1.57 (-2.89, -0.26) | 66% |
|  | 8000 mg/d | -6.9 (-9.01, -4.79) | 81% | -6.90 (-9.01, -4.79) | 81% |
|  | Total | -3.78 (-4.63, -2.92) | 81% | -3.51 (-4.43, -2.60) | 80% |
| **OGTT** | <1000 mg/d | -3.37 (-5.63, -1.10) | NA | NA | NA |
|  | 1000~2000 mg/d | -1.56 (-2.17, -0.94) | 0% | -1.60 (-2.43, -0.77) | 0% |
|  | 4000 mg/d | -1.35 (-2.47, -0.23) | NA | -1.35 (-2.47, -0.23) | NA |
|  | 8000 mg/d | -8.25 (-10.25, -6.25) | 54% | -8.25 (-10.25, -6.25) | 54% |
|  | Total | -4.89 (-6.27, -3.50) | 83% | -6.21 (-8.08, -4.33) | 85% |
| **TC** | <1000 mg/d | -1.71 (-2.44, -0.98) | 19% | -1.71 (-2.44, -0.98) | 19% |
|  | 1000~2000 mg/d | -1.87 (-2.73, -1.01) | 72% | -2.13 (-2.93, -1.33) | 60% |
|  | 4000 mg/d | -1.96 (-2.77, -1.16) | 0% | -1.96 (-2.77, -1.16) | 0% |
|  | 8000 mg/d | -3.45 (-4.29, -2.60) | 48% | -3.45 (-4.29, -2.60) | 48% |
|  | Total | -2.40 (-2.89, -1.90) | 63% | -2.47 (-2.93, -2.01) | 54% |
| **TG** | <1000 mg/d | -1.06 (-1.64, -0.48) | 0% | -1.06 (-1.64, -0.48) | 0% |
|  | 1000~2000 mg/d | -1.00 (-1.45, -0.55) | 28% | -1.10 (-1.58, -0.61) | 27% |
|  | 4000 mg/d | -0.99 (-1.66, -0.31) | 0% | -0.99 (-1.66, -0.31) | 0% |
|  | 8000 mg/d | -3.68 (-4.40, -2.95) | 24% | -3.68 (-4.40, -2.95) | 24% |
|  | Total | -1.90 (-2.39, -1.42) | 68% | -1.98 (-2.48, -1.48) | 67% |
| **HDL** | <1000 mg/d | 1.48 (0.66, 2.29) | 0% | 1.48 (0.66, 2.29) | 0% |
|  | 1000~2000 mg/d | 1.72 (0.80, 2.64) | 77% | 1.98 (1.06, 2.91) | 72% |
|  | 4000 mg/d | 1.65 (0.44, 2.87) | 59% | 1.65 (0.44, 2.87) | 59% |
|  | 8000 mg/d | 1.2 (0.70, 1.69) | 40% | 1.20 (0.70, 1.69) | 40% |
|  | Total | 1.43 (1.04, 1.82) | 57% | 1.50 (1.11, 1.89) | 53% |
| **LDL** | <1000 mg/d | -2.03 (-3.10, -0.96) | 27% | -2.03 (-3.10, -0.96) | 27% |
|  | 1000~2000 mg/d | -1.61 (-2.49, -0.73) | 75% | -1.87 (-2.71, -1.02) | 67% |
|  | 4000 mg/d | -1.52 (-3.14, 0.11) | 77% | -1.52 (-3.14, 0.11) | 77% |
|  | 8000 mg/d | -1.17 (-1.61, -0.72) | 26% | -1.17 (-1.61, -0.72) | 26% |
|  | Total | -1.41 (-1.81, -1.02) | 58% | -1.48 (-1.87, -1.10) | 52% |

**Supplementary table 2.** Results from sensitivity analysis. NA: not applicable.


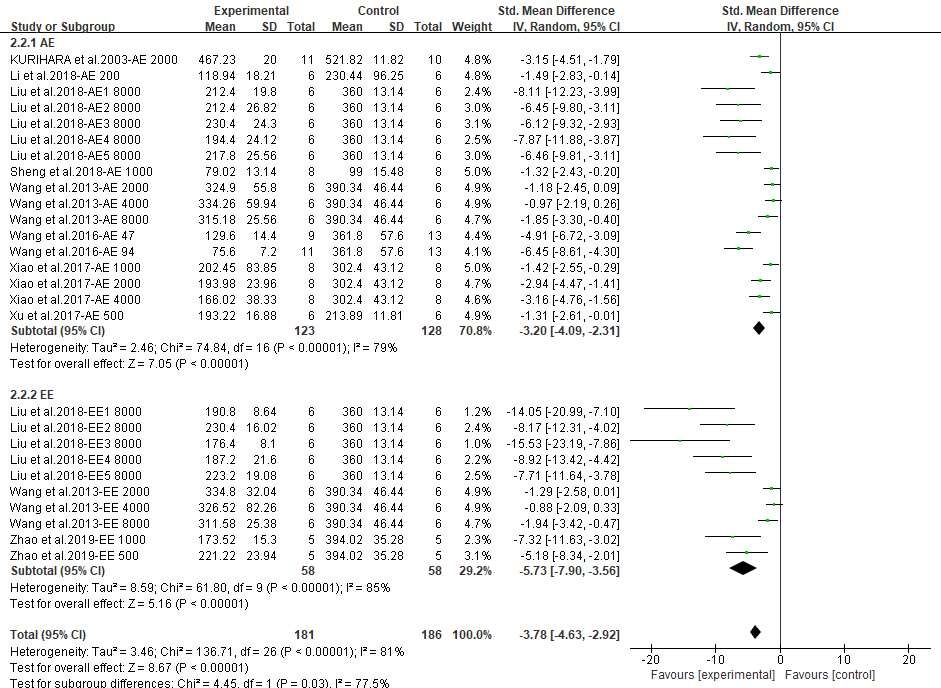


**Supplementary Figure 1.** Forest plot for the effect of *Cyclocarya paliurus* (CP) on blood glucose levels in animal models. Subgroup analysis was performed according to extracts.


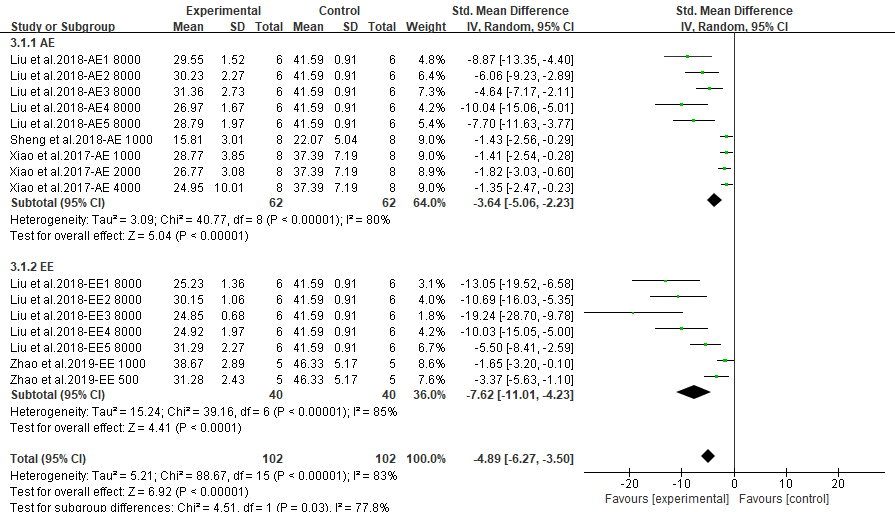


**Supplementary Figure 2.** Forest plot for the effect of *Cyclocarya paliurus* (CP) on area under curve of oral glucose tolerance test in animal models. Subgroup analysis was performed according


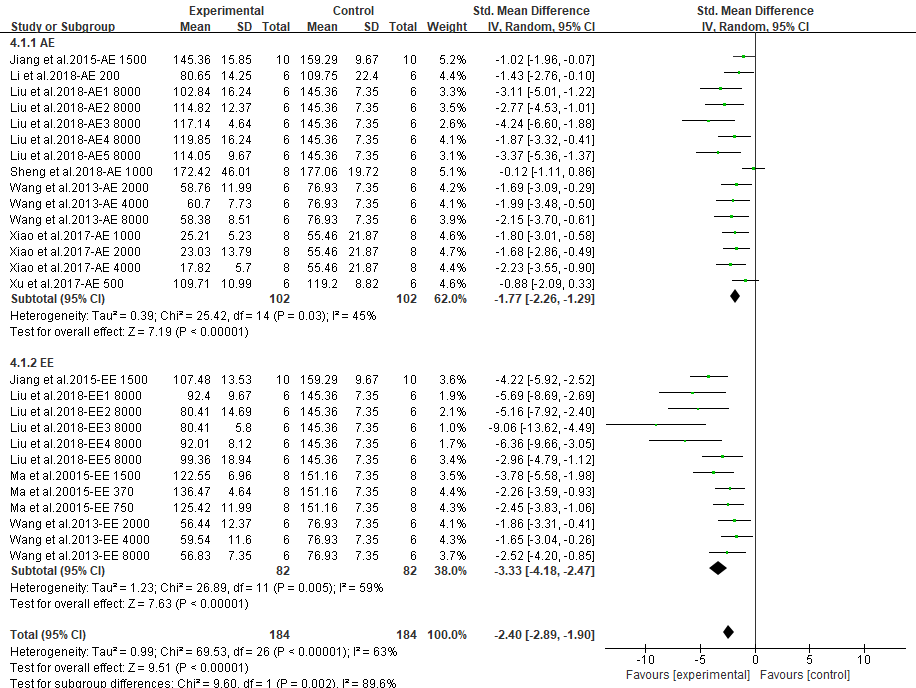


**Supplementary Figure 3.** Forest plot for the effect of *Cyclocarya paliurus* (CP) on total cholesterol levels in animal models. Subgroup analysis was performed according to extracts.


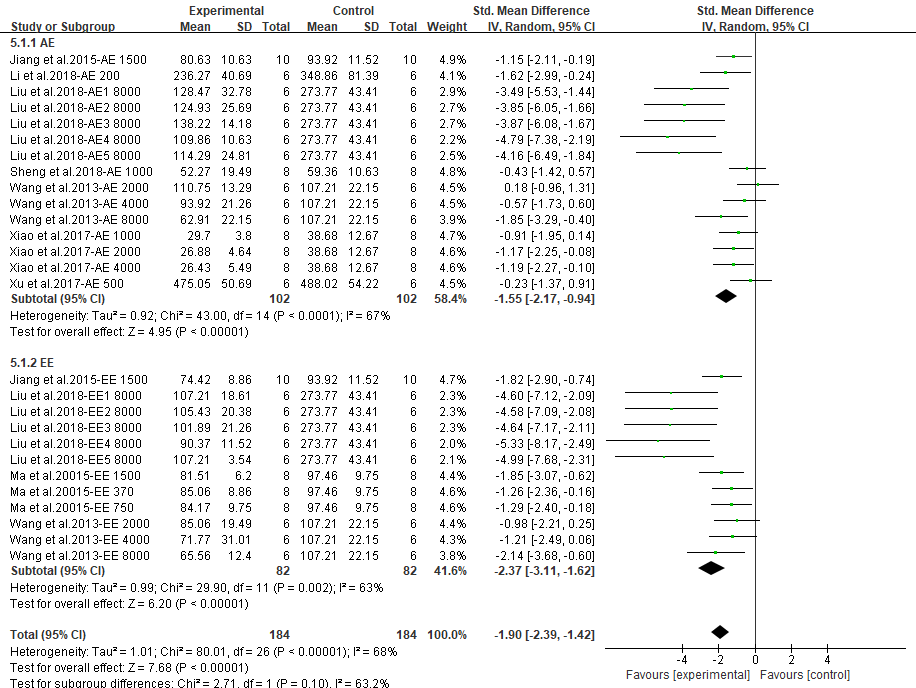


**Supplementary Figure 4.** Forest plot for the effect of *Cyclocarya paliurus* (CP) on triglyceride levels in animal models. Subgroup analysis was performed according to extracts.


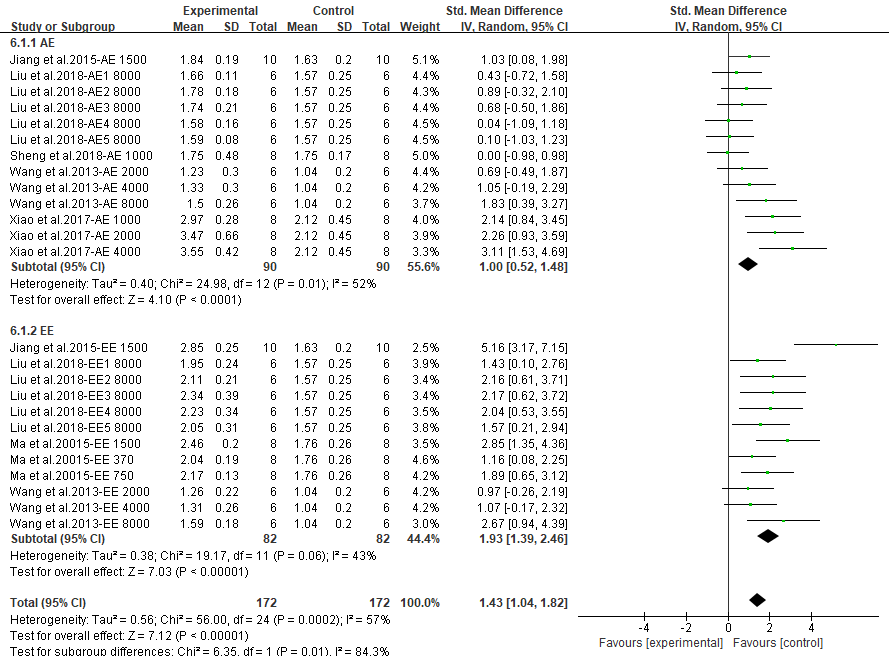


**Supplementary Figure 5.** Forest plot for the effect of *Cyclocarya paliurus* (CP) on high-density lipoprotein levels in animal models. Subgroup analysis was performed according to extracts.


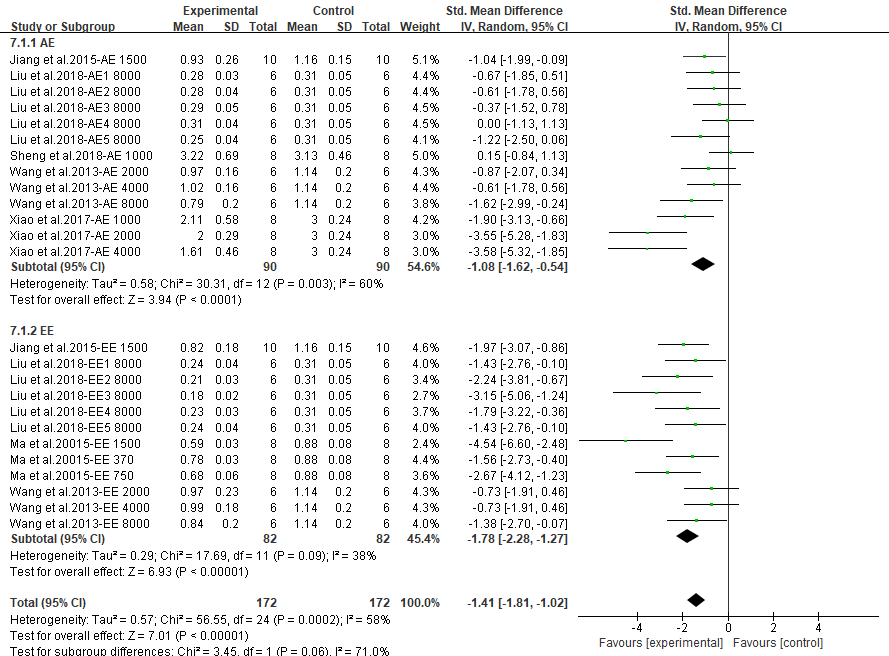


**Supplementary Figure 6.** Forest plot for the effect of *Cyclocarya paliurus* (CP) on low-density lipoprotein levels in animal models. Subgroup analysis was performed according to extracts.

**Supplementary Figure 7.** Trim and fill method on high-density lipoprotein (HDL).
